# Subthreshold *α*_2_-Adrenergic Activation Counteracts Glucagon-Like Peptide-1 Potentiation of Glucose-Stimulated Insulin Secretion

**DOI:** 10.1155/2011/604989

**Published:** 2010-12-27

**Authors:** Minglin Pan, Guang Yang, Xiuli Cui, Shao-Nian Yang

**Affiliations:** ^1^Endocrinology and Metabolic Diseases Laboratory, Tianjin Research Center of Basic Medical Sciences and Metabolic Diseases Hospital, Tianjin Medical University, Tianjin 300070, China; ^2^The Rolf Luft Research Center for Diabetes and Endocrinology, Karolinska Institutet, 171 76 Stockholm, Sweden

## Abstract

The pancreatic *β* cell harbors *α*_2_-adrenergic and glucagon-like peptide-1 (GLP-1) receptors on its plasma membrane to sense the corresponding ligands adrenaline/noradrenaline and GLP-1 to govern glucose-stimulated insulin secretion. However, it is not known whether these two signaling systems interact to gain the adequate and timely control of insulin release in response to glucose. The present work shows that the *α*_2_-adrenergic agonist clonidine concentration-dependently depresses glucose-stimulated insulin secretion from INS-1 cells. On the contrary, GLP-1 concentration-dependently potentiates insulin secretory response to glucose. Importantly, the present work reveals that subthreshold *α*_2_-adrenergic activation with clonidine counteracts GLP-1 potentiation of glucose-induced insulin secretion. This counteractory process relies on pertussis toxin- (PTX-) sensitive Gi proteins since it no longer occurs following PTX-mediated inactivation of Gi proteins. The counteraction of GLP-1 potentiation of glucose-stimulated insulin secretion by subthreshold *α*_2_-adrenergic activation is likely to serve as a molecular mechanism for the delicate regulation of insulin release.

## 1. Introduction

Glucose-stimulated insulin secretion plays an irreplaceable role in the control of glucose homeostasis since insulin is the only hormone capable of lowering blood glucose in the body [1–3]. This pancreatic endocrine hormone is packed in *β* cell secretory granules. These granules undergo exocytosis to release their insulin cargo into the bloodstream in response to elevated blood glucose levels [1–3]. Upon elevation of the plasma glucose level, the *β* cell efficiently takes up glucose through glucose transporters. Thereafter, subsequent glucose metabolism drastically raises the intracellular ATP level. The resultant rise in the ATP/ADP ratio closes ATP-sensitive K^+^ (K_ATP_) channels, causing depolarization of the plasma membrane. The membrane depolarization in turn opens voltage-gated Ca^2+^ (Ca_V_) channels, mediating Ca^2+^ influx. The consequent increase in cytosolic-free Ca^2+^ concentration ([Ca^2+^]_i_) triggers direct interactions between exocytotic proteins situated in the insulin-containing granule membrane and those localized in the plasma membrane. Eventually, the interaction between exocytotic proteins initiates the fusion of insulin-containing granules with the plasma membrane, that is, insulin exocytosis [1–3].

On top of the aforementioned consensus paradigm, glucose-stimulated insulin secretion is, in fact, regulated by complex neural mechanisms [4, 5]. It is well known that the autonomic nervous system innervates pancreatic islet cells where parasympathetic endings release a bunch of substances, for example, acetylcholine and vasoactive intestinal polypeptide, to potentiate glucose-stimulated insulin secretion [5, 6]. On the contrary, sympathetic terminals exocytose adrenergic and peptidergic transmitters to inhibit the insulin secretory process [4, 5]. Treatment with the main sympathetic transmitter noradrenaline fully shuts down insulin secretion from either islets or *β* cell aggregates perifused with high glucose [7, 8]. Mechanistically noradrenaline acts on *α*_2_ receptors coupled to pertussis toxin- (PTX-) sensitive Gi proteins in *β* cells, reducing glucose-stimulated insulin secretion through inhibition of intracellular cAMP formation, Ca_V_ channels, glucose metabolism, and the exocytotic machinery as well as elevation of K_ATP_ channel activity [4, 5].

Glucose-stimulated insulin secretion is subjected not only to the complex neural regulation, but also to various different types of hormonal regulation [9–16]. The islet *β* cell is able to sense its own released molecules, such as zinc and ATP, and hormones released from its neighboring cells to autocrinally and paracrinally regulate insulin secretion in response to glucose stimulation [13–18]. A number of systemic hormones impinge on islet *β* cells to coordinate insulin secretory response to glucose [9–12, 19]. A group of gastrointestinal hormones has long attracted a great deal of attention and categorized as incretins due to their stimulatory action on glucose-stimulated insulin secretion [9–12]. One of the most important incretin hormones is glucagon-like peptide-1 (GLP-1), which is secreted from intestinal L-cells into the bloodstream after a meal [9–12]. Upon encounter with *β* cells, this incretin binds to Gs protein-coupled receptors on these cells, resulting in activation of adenylyl cyclases, Ca_V_ channels, glucose metabolism, and the exocytotic machinery as well as inhibition of K_ATP_ channels [9–12, 20–22]. As consequence of these events, potentiation of glucose-stimulated insulin secretion occurs [9–12, 20–22].

Although either noradrenergic or GLP-1 signaling system in the regulation of glucose-stimulated insulin secretion has been clarified, it is not known whether these two signaling systems interact to gain adequate and timely insulin release in response to glucose stimulation [4, 5, 9–12, 20–22]. In the present work, we describe that subthreshold *α*_2_-adrenergic activation counteracts glucagon-like peptide-1 potentiation of glucose-stimulated insulin secretion in a PTX-sensitive Gi protein-dependent manner.

## 2. Materials and Methods

### 2.1. Cell Culture

INS-1 cells were cultivated in RPMI 1640 medium (Invitrogen, Carlsbad, CA) supplemented with the following additives: 10% fetal bovine serum, 2 mM L-glutamine, and 100 U/100 *μ*g/ml penicillin/streptomycin, 10 mM N-[2-hydroxyethyl piperazine-N^'^-2-ethanesulfonic acid (HEPES), 1 mM sodium pyruvate, and 50 *μ*M *β*-mercaptoethanol (Invitrogen). Briefly, the cells at about 70% confluency were trypsinized. The resultant cell suspension was seeded into 24-well cell culture plates. The cells were maintained at 37°C in a humidified 5% CO_2_ incubator. They were grown to approximately 70% confluence and then subjected to analysis of insulin secretion.

### 2.2. Static Insulin Secretion

Approximately 70% confluent INS-1 cells in 24-well plates were used for insulin secretion experiments. The cells were kept at 37°C in a humidified 5% CO_2_ incubator during the course of an experiment except when their bath solutions need to be changed. The experiments were carried out in Krebs-Ringer bicarbonate HEPES buffer (KRBH) consisting of (in mM) 140NaCl, 3.6KCl, 1.5CaCl_2_, 0.5MgSO_4_, 0.5NaH_2_PO_4_, 2NaHCO_3_, 10HEPES, 0.1% bovine serum albumin (BSA), pH 7.4. First, the cells were rinsed with glucose-free KRBH and then maintained in the same buffer for 2 h. Thereafter, they were rinsed and preincubated with glucose-free KRBH for 30 min. To characterize the concentration-response relationships of the *α*_2_-adrenergic agonist clonidine and the incretin GLP-1 as well as interactions between noradrenergic and GLP-1 signaling systems, the cells were rinsed and incubated with 3 or 11 mM glucose KRBH containing different concentrations of clonidine and/or GLP-1 for 30 min. Clonidine and/or GLP-1 were applied simultaneously with glucose. To determine a possible dependence of *α*_2_-adrenoceptor regulation of GLP-1 receptors on PTX-sensitive Gi proteins, the cells were pretreated with 100 ng/ml PTX in RPMI 1640 medium for 18 h. Subsequently, the toxin medium was removed. The cells were rinsed and incubated with glucose-free KRBH as described above and subjected to incubations with 3 or 11 mM glucose KRBH containing different concentrations of clonidine and/or GLP-1 for 30 min. Finally, the treatments with the different reagents were stopped by putting the culture plates on ice. Supernatants were carefully aspirated from each well to prepare samples for insulin quantification. The samples were centrifuged at 1000 × g for 3 min to remove detached cells and stored at −20°C until insulin immunoassay was performed.

### 2.3. Radioimmunoassay

A standard insulin immunoassay was used to evaluate static insulin secretion from INS-1 cells subjected to different treatments [23, 24]. Briefly, duplicate samples were measured. The calibration curve was constructed from insulin standard at 5, 10, 20, 40, 80, and 160 mIU/L. Radioactivity was counted by a *γ*-counter.

### 2.4. Statistical Analysis

All data are presented as mean ± SEM. Statistical significance was evaluated by one-way ANOVA, followed by least significant difference (LSD) test. The significance level was determined at both the 0.05 and 0.01 levels.

## 3. Results

### 3.1. Clonidine Concentration-Dependently Inhibits Glucose-Stimulated Insulin Secretion

**Figure 1:**
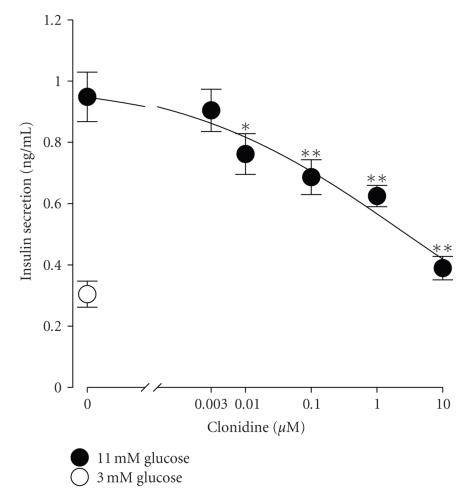
The *α*_2_-adrenergic agonist clonidine concentration-dependently depresses glucose-stimulated insulin secretion from INS-1 cells. Static insulin secretion was performed with cells subjected to stepwise elevation of glucose concentration from 3 to 11 mM for 30 min in the absence or presence of clonidine and determined by a standard insulin radioimmunoassay. Cells exposed to 11 mM glucose (closed circle at the far left) released significantly more insulin than those to 3 mM glucose (open circle) (*n* = 6, *P* < .01). In the concentration range of 0.003–10 *μ*M, clonidine produced a concentration-dependent inhibition of insulin release induced by 11 mM glucose. The inhibition became statistically significant at 0.01 *μ*M clonidine (*n* = 6, *P* < .05) and was statistically significant at higher clonidine concentrations (*n* = 6, *P* < .01). The subthreshold and ED_50_ concentration of clonidine were calculated to be 0.003 and 4 *μ*M, respectively. In this and all other figures, data are presented as means ± SEM. Statistical significance was evaluated by one-way ANOVA, followed by least significant difference (LSD) test. *P < .05 and **P < .01 versus 11 mM glucose-treated group.

To determine the concentration-response relationship of clonidine inhibition of glucose-stimulated insulin secretion, we examined the effect of 30 min incubation with clonidine at concentrations ranging from 0.003 to 10 *μ*M on insulin release from INS-1 cells challenged with 11 mM glucose. As shown in Figure 1, incubation with 11 mM glucose for 30 min resulted in a significant insulin secretion as compared with that with 3 mM glucose (*n* = 6, *P* < .01). This confirms that the cells used in this set of experiments reliably responded to such stimulation to secrete an appreciable amount of insulin. We therefore adopted this sufficient and reliable stimulation to test for the effect of clonidine on glucose-stimulated insulin secretion. Figure 1 shows that in the concentration range of 0.003–10 *μ*M, clonidine concentration-dependently inhibited insulin release from INS-1 cells exposed to 11 mM glucose. The effect became statistically significant when clonidine concentration reached 0.01 *μ*M and higher (*n* = 6, *P* < .05 at 0.01 *μ*M, *P* < .01 at 0.1, 1 and 10 *μ*M). The subthreshold and ED_50_ concentration of clonidine were estimated to be 0.003 and 4 *μ*M, respectively.

### 3.2. Glucagon-Like Peptide-1 Concentration-Dependently Stimulates Glucose-Stimulated Insulin Secretion

**Figure 2:**
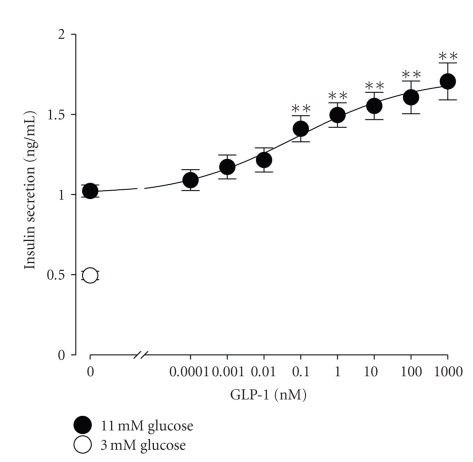
The incretin GLP-1 concentration-dependently potentiates glucose-stimulated insulin secretion from INS-1 cells. A stepwise increase in glucose concentration from 3 to 11 mM for 30 min was used to induce static insulin secretion from cells exposed to different concentrations of GLP-1. Released insulin was measured by a standard insulin radioimmunoassay. 11 mM glucose incubation (closed circle at the far left) caused a significant insulin secretion from cells preincubated with 3 mM glucose (*n* = 6, *P* < .01 versus 3 mM glucose-treated group represented by an open circle). GLP-1 at concentrations ranging from 0.0001 to 1000 nM potentiated glucose-stimulated insulin secretion in a concentration-dependent manner. GLP-1 at concentrations of 0.1 nM and higher significantly enhanced insulin release from cells stimulated with 11 mM glucose (*n* = 6, *P* < .01 versus 11 mM glucose-treated group). The subthreshold and ED_50_ concentration of GLP-1 were estimated to be 0.01 and 0.1 nM, respectively. **P < .01 versus 11 mM glucose-treated group.

To reveal the concentration-response relationship of GLP-1 potentiation of glucose-stimulated insulin secretion, we evaluated the insulin secretory response of INS-1 cells stimulated with 11 mM glucose for 30 min in the presence of GLP-1 in the concentration range 0.0001 to 1000 nM.  Figure 2 shows that 11 mM glucose treatment for 30 min produced a significant increase in insulin secretion in comparison with 3 mM glucose treatment (*n* = 6, *P* < .01). This validates that the glucose responsiveness of the cells employed in this set of experiments. As illustrated in Figure 2, GLP-1 in the concentration range 0.0001 to 1000 nM significantly potentiated insulin release induced by 11 mM glucose in a concentration-dependent manner. The statistically significant potentiation occurred when GLP-1 concentration was raised to 0.1 nM and higher (*n* = 6, *P* < .01). A concentration of 0.01 nM was considered as the subthreshold concentration of GLP-1 on its potentiation of glucose-stimulated insulin secretion. The ED_50_ concentration of GLP-1 for potentiating the insulin secretory response to glucose was calculated to be 0.1 nM.

### 3.3. Subthreshold Clonidine Suppresses the Stimulatory Effect of Glucagon-Like Peptide-1 on Glucose-Stimulated Insulin Secretion

The pancreatic *β* cell is equipped with both the *α*_2_-adrenergic receptor and the GLP-1 receptor which are impinged by the sympathetic transmitter adrenaline/noradrenaline and the incretin hormone GLP-1, respectively [4, 5, 9–12, 20–22]. Both of these systems critically regulate glucose-stimulated insulin secretion [4, 5, 9–12, 20–22]. This inevitably raises the question whether they are insulated from each other or one cross-talks with the other in pancreatic *β* cells. To tackle this issue, we examined how subthreshold *α*_2_-adrenergic activation affects GLP-1 potentiation of glucose-stimulated insulin secretion.

**Figure 3:**
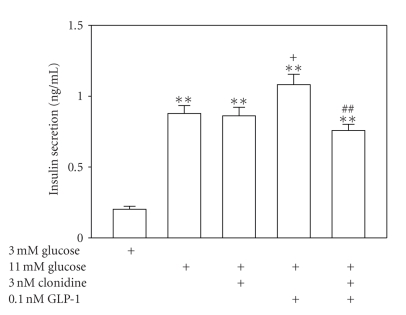
Subthreshold *α*_2_-adrenergic activation with clonidine counteracts GLP-1 potentiation of glucose-stimulated insulin secretion. A standard insulin radioimmunoassay was employed to examine the insulin secretory response of cells treated with the subthreshold concentration of clonidine, the ED_50_ concentration of GLP-1 and their combinations following a stepwise stimulation with glucose from 3 to 11 mM for 30 min. Treatment with 11 mM glucose caused a significant insulin secretion as compared with that with 3 mM glucose (*n* = 10, *P* < .01). Cells incubated with clonidine at the subthreshold concentration 3 nM and control cells displayed similar insulin secretory responses to 11 mM glucose (*n* = 10, *P* > .05). However, cells treated with GLP-1 at the ED_50_ concentration 0.1 nM displayed significantly enhanced insulin secretion in comparison with untreated cells following 11 mM glucose stimulation (*n* = 10, *P* < .05). Furthermore, cells exposed to the ED_50_ concentration of GLP-1 plus the subthreshold concentration of clonidine exhibited significantly less insulin secretion than cells treated with the ED_50_ concentration of GLP-1 alone following 11 mM glucose stimulation (*n* = 10, *P* < .01). Control cells and cells treated either with the ED_50_ concentration of GLP-1 plus the subthreshold concentration of clonidine or the subthreshold concentration of clonidine alone released similar amounts of insulin in response to 11 mM glucose (*n* = 10, *P* > .05). **P < .01 versus 3 mM glucose-treated group, ^+^ *P* < .05 versus 11 mM glucose-treated group. ^##^ *P* < .01 versus the ED50 concentration of GLP-1 plus the subthreshold concentration of clonidine group subjected to 11 mM glucose incubation.

Validation of the capacity of the cells applied in this set of experiments to release insulin in response to glucose was likewise performed. As illustrated in Figure 3, treatment with 11 mM glucose for 30 min gave rise to a significant insulin release as compared with that with 3 mM glucose (*n* = 10, *P* < .01). As expected, cells exposed to clonidine at the subthreshold concentration 3 nM did not alter their insulin secretory response to 11 mM glucose (*n* = 10, *P* > .05 versus group subjected to only 11 mM glucose stimulation) (Figure 3). In contrast, cells treated with GLP-1 at the ED_50_ concentration 0.1 nM following 11 mM glucose stimulation released significantly more insulin than cells subjected to only 11 mM glucose stimulation (*n* = 10, *P* < .05). Importantly, cells incubated with the ED_50_ concentration of GLP-1 plus the subthreshold concentration of clonidine secreted significantly less insulin than cells treated with the ED_50_ concentration of GLP-1 alone following 11 mM glucose stimulation (*n* = 10, *P* < .01) (Figure 3). The insulin secretory response to 11 mM glucose was very similar among group treated with the ED_50_ concentration of GLP-1 plus the subthreshold concentration of clonidine, group treated with the subthreshold concentration of clonidine alone, and untreated group (*n* = 10, *P* > .05) (Figure 3). The data demonstrate that the subthreshold concentration of clonidine completely counteracted the potentiation of glucose-stimulated insulin secretion by the ED_50_ concentration of GLP-1.

### 3.4. Counteraction of Glucagon-Like Peptide-1 Potentiation of Glucose-Stimulated Insulin Secretion by Clonidine Relies on Pertussis Toxin-Sensitive Gi Proteins

Multiple intracellular signaling events, such as decreases in cAMP production, Ca_V_ channel activity, glucose metabolism, and exocytotic capacity as well as an increase in K_ATP_ conductance occur upon activation of *α*_2_-adrenergic receptors on the *β* cell to depress glucose-stimulated insulin secretion [4, 5]. All these events are dependent on the PTX-sensitive Gi protein that is an immediate mediator for *α*_2_-adrenergic activation [4, 5]. This made us wonder if counteraction of GLP-1 potentiation of glucose-stimulated insulin secretion by clonidine relies on PTX-sensitive Gi proteins. To circumvent this issue, we evaluated if PTX-mediated inactivation of Gi proteins could prevent counteraction of GLP-1 potentiation of glucose-stimulated insulin secretion by subthreshold *α*_2_-adrenergic activation.

**Figure 4:**
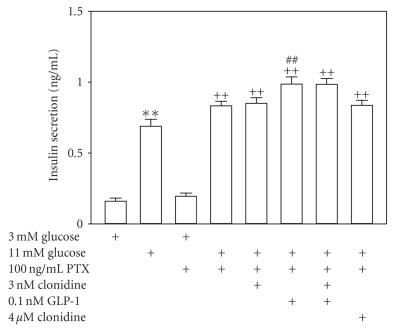
Uncoupling of Gi proteins with PTX prevents counteraction of GLP-1 potentiation of glucose-stimulated insulin secretion by clonidine. Cells pretreated with 100 ng/ml PTX for 18 h and control cells were subjected to analysis of static insulin secretion induced by 11 mM glucose for 30 min in the absence or presence of the subthreshold concentration and ED_50_ concentration of clonidine, the ED_50_ concentration of GLP-1, and their combinations. Insulin secretion was evaluated by a standard insulin radioimmunoassay. Both control and PTX-pretreated cells well responded to 30 min stimulation with 11 mM glucose (*n* = 11, *P* < .01 versus corresponding 3 mM glucose-treated groups). PTX-pretreated cells released significantly more insulin than control cells following 11 mM glucose stimulation (*n* = 11, *P* < .01). Neither the subthreshold concentration (3 nM) nor the ED_50_ concentration (4 *μ*M) of clonidine altered glucose-stimulated insulin secretion in PTX-pretreated cells (*n* = 11, *P* > .05 versus PTX-pretreated group subjected to only 11 mM glucose stimulation). In contrast, GLP-1 at the ED_50_ concentration of 0.1 nM induced a significant potentiation of glucose-induced insulin release from PTX-pretreated cells (*n* = 11, *P* < .01 versus PTX-pretreated group subjected to only 11 mM glucose stimulation). Intriguingly, glucose-stimulated insulin secretion from PTX-pretreated cells incubated with the ED_50_ concentration of GLP-1 alone did not significantly differ from that from those subjected to incubation with the ED_50_ concentration of GLP-1 plus the subthreshold concentration of clonidine (*n* = 11, *P* > .05). Clonidine at the subthreshold concentration could no longer influence potentiation of glucose-stimulated insulin secretion by the ED_50_ concentration of GLP-1 in PTX-pretreated cells. **P < .01 versus 3 mM glucose-treated group without PTX pretreatment, ^++^ *P* < .01 versus 11 mM glucose-treated group without PTX pretreatment or PTX-pretreated group subjected to 3 mM glucose incubation, ^##^ *P* < .01 versus PTX-pretreated group subjected to 11 mM glucose stimulation.

The cells used in this set of experiments were proved to be quite sensitive to glucose with regard to their insulin secretory responsiveness.  Figure 4 shows that both control and PTX-pretreated cells released a significant amount of insulin when glucose concentration was raised from 3 to 11 mM (*n* = 11, *P* < .01). Cells pretreated with 100 ng/ml PTX in for 18 h secreted significantly more insulin than cells without PTX pretreatment following 11 mM glucose stimulation (*n* = 11, *P* < .01) (Figure 4). Clonidine at both the subthreshold concentration 3 nM and the ED_50_ concentration 4 *μ*M had no effect on insulin secretory response to 11 mM glucose in PTX-pretreated cells (*n* = 11, *P* > .05 versus PTX-pretreated group subjected to only 11 mM glucose stimulation) (Figure 4). However, GLP-1 at the ED_50_ concentration 0.1 nM significantly enhanced insulin secretion from PTX-pretreated cells following 11 mM glucose stimulation (*n* = 11, *P* < .01 versus PTX-pretreated group subjected to only 11 mM glucose stimulation) (Figure 4). Most importantly, the subthreshold concentration of clonidine was unable to counteract potentiation of glucose-stimulated insulin secretion by the ED_50_ concentration of GLP-1 in PTX-pretreated cells (*n* = 11, *P* > .05 versus PTX-pretreated group treated with 11 mM glucose plus 0.1 nM GLP-1) (Figure 4). The data reveal that counteraction of GLP-1 potentiation of glucose-stimulated insulin secretion by clonidine relies on PTX-sensitive Gi proteins.

## 4. Discussion

Glucose homeostasis critically relies on the complex regulation of glucose-stimulated insulin secretion by autonomic impulses and humoral inputs to the pancreatic *β* cell [4, 5, 9–16]. *α*_2_-Adrenergic and GLP-1 receptors on the pancreatic *β* cell transduce signals from their corresponding ligands adrenaline/noradrenaline and GLP-1 to control glucose-induced insulin release [4, 5, 9–12]. The present work confirms that the *α*_2_-adrenergic agonist clonidine and the incretin GLP-1 concentration-dependently inhibits glucose-induced insulin release at concentration ranges similar to those employed in previous studies [8, 25, 26]. Furthermore, it also estimates the subthreshold and ED_50_ concentration of theses two agonists. These parameters are critical for examination of the counteraction of GLP-1 potentiation of glucose-stimulated insulin secretion by subthreshold *α*_2_-adrenergic activation.

Most importantly, the present study shows for the first time that insulin-secreting INS-1 cells exposed to the ED_50_ concentration of GLP-1 together with the subthreshold concentration of clonidine release significantly less insulin than cells treated with the ED_50_ concentration of GLP-1 alone following glucose stimulation. Furthermore, it also uncovers that the antagonistic interaction of the *α*_2_-adrenergic signaling system with the GLP-1 signaling system critically depends on PTX-sensitive Gi proteins. These findings provide evidence that *α*_2_-adrenergic or GLP-1 signaling system does not operate independently, but instead the former effectively antagonizes the latter to enable the pancreatic *β* cell to appropriately execute its unique function glucose-stimulated insulin secretion. In fact, interactions between G protein-coupled receptor signaling pathways have been intensively investigated in other cell types and neurons in particular [27–33]. Such interactions rely on multilevel mechanisms [27–33]. They occur at the receptor level due to receptor heterodimerization, which is either G protein-dependent or -independent [27–30]. The heterodimerization is able to alter the ligand binding affinity and/or signal transduction efficacy of dimerized receptors [27–30, 32, 33]. Interactions between G protein-coupled receptor signaling pathways can also bypass the receptor level and come about downstream of receptors as a result of crosstalk between receptor signaling cascades [31]. In general, these well-characterized mechanisms are applicable to the counteraction of GLP-1 potentiation of glucose-stimulated insulin secretion by subthreshold *α*_2_-adrenergic activation in the pancreatic *β* cell. The in-depth mechanisms whereby the *α*_2_-adrenergic signaling system antagonizes the GLP-1 signaling system in the pancreatic *β* cell remain to be characterized.

There is no doubt that the healthy body requires the efficient amount of insulin to remove extra glucose from the blood stream into body cells most of the time. However, the healthy body needs less insulin to boost blood glucose levels in some circumstances, such as stress, exercise, low blood glucose, and other environmental challenges. The counteraction of GLP-1 potentiation of glucose-stimulated insulin secretion by subthreshold *α*_2_-adrenergic activation definitely fits in with these circumstances where sympathetic activity is elevated [34]. It adds a new level of complexity to the classical paradigm for the regulation of glucose-evoked insulin release. Under certain pathological conditions, for example, diabetes, hypertension, obesity, and aging, sympathetic activity and/or expression of *α*_2_-adrenergic receptors in the *β* cell significantly increase [35–39]. Increases in sympathetic activity and/or expression of *α*_2_-adrenergic receptors in the *β* cell likely exaggerate the antagonistic interaction of the *α*_2_-adrenergic signaling system with the GLP-1 signaling system in the pancreatic *β* cell to provoke and aggravate diabetes [35–39].

## 5. Conclusions

*α*_2_-Adrenergic receptors and GLP-1 receptors on insulin-secreting INS-1 cells transduce signals from their corresponding ligands clonidine and GLP-1 to govern glucose-induced insulin release. Importantly, the former also interacts with the latter to brake potentiation of glucose-induced insulin release by the latter. In fact, subthreshold *α*_2_-adrenergic activation is enough to counteract GLP-1 potentiation of glucose-induced insulin secretion in a PTX-sensitive Gi protein-dependent fashion. Such a counteraction is able to serve as a molecular mechanism for the delicate control of insulin release in the healthy body. Most likely, this counteractory process is exaggerated to provoke and aggravate diabetes since obesity, aging, and diabetes are highly associated with elevated sympathetic activity [35–39].
