# Erratum to “Subthreshold *α*_2_-Adrenergic Activation Counteracts Glucagon-Like Peptide-1 Potentiation of Glucose-Stimulated Insulin Secretion”

**DOI:** 10.1155/2011/319202

**Published:** 2011-02-08

**Authors:** Minglin Pan, Guang Yang, Xiuli Cui, Shao-Nian Yang

**Affiliations:** ^1^Endocrinology and Metabolic Diseases Laboratory, Tianjin Research Center of Basic Medical Sciences and Metabolic Diseases Hospital, Tianjin Medical University, Tianjin 300070, China; ^2^The Rolf Luft Research Center for Diabetes and Endocrinology, Karolinska Institutet, 171 76 Stockholm, Sweden

There are three errors in the original paper. First, the last sentence of the legend for Figure 3 should have read “^##^ *P* < .01 versus group subjected to treatment with the ED_50_ concentration of GLP-1 and stimulation with 11 mM glucose.” Second, the word “inhibits” in the third line of the right column on page 5 should have read “regulate.” Third, the typo “LD” in Reference 23 on page 6 should have read “L_D_”. We apologize to readers for these errors.

